# Annotations

**Published:** 1902-12-13

**Authors:** 


### Annotations.

### Plague in San Francisco.

Much as there is to admire in American institutions, there can be no doubt that the large degree of independence which is enjoyed by the different States renders it very difficult to carry out any comprehensive sanitary policy. During the past two or three years rumours have again and again obtained currency as to the existence of plague in San Francisco, rumours which have been quickly met by positive assurances as to the healthiness of that city. It now appears, however, that bubonic plague has been present in San Francisco all the time. According to the American papers, over 30 cases have been reported in the last seven months, and exactly the same number of deaths have taken place. This, as we need hardly say, puts a very grave aspect upon the affair. Plague is not so fatal a disease as all that, and our experience in India and elsewhere has repeatedly enforced the lesson that whenever the mortality equals, or nearly equals, the notifications, the explanation is that cases are being hidden, only those in which death appears inevitable being reported to the authorities. Such a state of affairs is always a difficult one to deal with, for it shows the existence of a strong and widespread public feeling against sanitary control. How much greater, then, is the danger when, as the result of connivance between the State and city authorities, the fact of the presence of the disease is suppressed, and a focus of infection is allowed to take root on American soil.

### The Propagation of the Unfit.

The present is said to be a scientific age ; in some respects this is true. But it is also a period when charlatanism abounds, superficiality dominates, and science, falsely so-called, seeks to allure the very elect. We are surprised, however, to find the pages in a recent issue of our distinguished contemporary *The Academy and Literature* disfigured by a misleading article on "The Increase of the Unfit." The writer dogmatically announces that the number of insane persons in the community has been increasing for the last fifty years, and says cancer during the same period has doubled the number of its victims. Tuberculosis, we are also told, would probably have done the same were it not that improvements in treatment have led to the cure of slight cases in their early stage, and to the prolongation of life in the more severe. And it is suggested that this calamitous state of affairs probably arises from admixture of alien blood ; "we should recognise the fact that the growing contamination of the nation's blood should be checked at all hazards." The cause of tuberculosis we are informed is almost certainly a "bacterium," and it and cancer and insanity are "transmitted by descent." And the remedy forsooth is "the prevention of the marriage of persons having an hereditary taint." But since humanity is perverse it must be caught by guile, and parties proposing to marry are to be compelled to produce evidence that they have insured their lives, and thus may presumably be considered free from hereditary disease. The writer seems to be ignorant of the fact that not a few companies take "doubtful" lives, and even some accept applicants without any medical examination at all. And there is a growing disposition to discard medical examination in the selection of candidates for insurance. But all these points are neglected, and it is presumed that assurance societies will always be guided by medical considerations. And so the superficial dreamer of *The Academy* thinks that if the required statute were passed now our children might see a nation of Englishmen sane and healthy in the sense that they would be free from any hereditary tendency towards some of the most terrible scourges that can afflict the human race. The writer laments that his voice is as one that crieth in the wilderness. We wish it were, for we fear that such teaching is doing much to hinder true progress, and instead of assisting in the solution of mysteries of being is only too effectually setting back the clock.

### Abandoned Children.

"It isn't the women who take a number of children for regular payments that want looking after so much. They need watching, certainly ; for they are ignorant, and often don't give the children the proper kind of food. But in the main they are honest. It is those who 'adopt' them, as they call it, for a five-pound note—and no after-claim. They are the bad ones. You may be sure that child has got to die before the money given can be spent on it." This is the expressed opinion of a medical woman who inspects baby farms under the Infant Life Protection Act, and it is only too well borne out by the evidence given in recent cases. The possibility of abuse shows that the Infant Life Protection Act does not do all the work it is meant to do. One case may be discovered, but who is there who doubts that similar cases are to be found in many places ? It is the "adoption" and the lump sum paid down that works the mischief. When father or mother is paying weekly for the child, there is some guarantee that some inquiry will be made as to whether it still lives or not. People want value for their money. But when parent or friend has paid all that is to be paid, when all connected with its undesired advent have washed their hands of the child, the nurse, or foster-mother, or by whatever name she calls herself, is often justified in thinking that no further inquiry will be made, and even that the most welcome news that could be given to all concerned would be that the child is dead. That being so, the wonder is not that so many illegitimate children die, but that so many survive. If abandoned, they may have the luck, as one of Gale's nurslings had, to be found in time and sent to the workhouse. But there is the equal chance of their lying helpless and exposed until they die. The prime responsibility for this rests, after all, not with the hireling who takes the child and conceals its parentage from the world, but with the parents who thus get rid of their offspring. We have, rightly, a high ideal of womanly chastity, but it is unfortunate if, through that "homage which vice pays to virtue"—hypocrisy—this ideal leads to the neglect of a virtue as sacred—the duty which a mother owes to her child.

